# An Insight of Deep Learning Based Demand Forecasting in Smart Grids

**DOI:** 10.3390/s23031467

**Published:** 2023-01-28

**Authors:** Javier Manuel Aguiar-Pérez, María Ángeles Pérez-Juárez

**Affiliations:** Departamento de Teoría de la Señal y Comunicaciones e Ingeniería Telemática, Universidad de Valladolid, ETSI Telecomunicación, Paseo de Belén 15, 47011 Valladolid, Spain

**Keywords:** demand forecasting, load forecasting, demand response, forecasting horizon, smart grid, smart environment, Deep Learning, Long Short-Term Memory networks, Convolutional Neural Networks

## Abstract

Smart grids are able to forecast customers’ consumption patterns, i.e., their energy demand, and consequently electricity can be transmitted after taking into account the expected demand. To face today’s demand forecasting challenges, where the data generated by smart grids is huge, modern data-driven techniques need to be used. In this scenario, Deep Learning models are a good alternative to learn patterns from customer data and then forecast demand for different forecasting horizons. Among the commonly used Artificial Neural Networks, Long Short-Term Memory networks—based on Recurrent Neural Networks—are playing a prominent role. This paper provides an insight into the importance of the demand forecasting issue, and other related factors, in the context of smart grids, and collects some experiences of the use of Deep Learning techniques, for demand forecasting purposes. To have an efficient power system, a balance between supply and demand is necessary. Therefore, industry stakeholders and researchers should make a special effort in load forecasting, especially in the short term, which is critical for demand response.

## 1. Introduction

Electricity cannot be easily stored for future supply, unlike other commodities such as oil. This means that electricity must be distributed to the consumers immediately after its production. The distribution of electricity to final users has been done with the help of the traditional electrical grid (see definition in Table 1) which allows the delivery of electricity from producers to consumers. To achieve that goal, it connects the electricity generating stations and the transmission lines that deliver the electricity to the final users. Traditional electrical grids vary in size. When these grids started to expand, controlling them became a very complex and difficult task. Additionally, demand forecasting (see definition in Table 1) has not traditionally been considered.

In this context, the concept of the smart grid (see definition in Table 1) arises and starts to play an important role. This concept has been exhaustively reviewed in the literature (e.g., [1,2,3,4]). Smart grids provide a two-way communication between consumers and suppliers. Smart grids add hardware and software to the traditional electrical grid to provide it with an autonomous response capacity to different events that can affect the electrical grid. The final objective is to achieve an optimal daily operational efficiency for the electrical power delivery. In [4], the authors define “smart grid” as a new form of electricity network that offers self-healing, power-flow control, energy security and energy reliability using digital technology. In [2], the authors highlight that the concept of the smart grid is transforming the traditional electrical grid by using different types of advanced technology. According to these authors, this concept integrates all the elements that are necessary to generate, distribute, and consume energy efficiently and effectively. In [5], the authors emphasize that the smart grid concept emerged to make the traditional electrical grid more efficient, secure, reliable, and stable, and to be able to implement demand response (see definition in Table 1).

The smart grid paradigm allows consumers to find out their energy usage patterns. Consequently, consumers can control their consumption and use energy more efficiently. In the implementation of the smart grid concept, demand response—for both household and industrial purposes—plays an important role. Another useful tool is load forecasting (see definition in Table 1). In [6] the authors mark the importance of this concept in the context of smart grids, as forecasting the electricity needed to meet demand allows power companies to better balance demand and supply. Power companies are especially interested in achieving accurate forecasts for the next 24 h, which is called load profile (see definition in Table 1).

In addition, in recent years, the increased demand for electricity at certain times of the day has created several problems. Load forecasting is especially important during peak hours. Demand response encourages customers to offload non-essential energy consumption during these peak hours.

To face load forecasting challenges, it is necessary to use modern data-driven techniques. Indeed, the incorporation of new technologies, such as Big Data, Machine Learning, Deep Learning, and the Internet of Things (IoT), has upgraded the smart grid concept to another level, as these technologies allow for improved demand forecasting and automated demand response.

This paper provides an insight into the importance of demand forecasting and important related factors in the context of smart grids, as well as the possibility of using data-driven techniques for this purpose. More specifically, the authors focus on Deep Learning techniques, as it has emerged as a good option for the implementation of demand forecasting in the context of smart grids. The paper collects some experiences of using different Deep Learning techniques in the energy domain for forecasting purposes. An efficient power system must take demand response into account. Additionally, accurate load forecasting, especially in the short term, is essential, which is why industry stakeholders and researchers are putting special efforts into it.

Table 1 defines some keywords related to the topic of this paper.

The remainder of this paper is organized as follows. Section 2 presents the reasons why demand forecasting is important in the context of smart grids. Section 3 describes the most important factors in relation to demand forecasting. Section 4 presents the different possible classifications of demand forecasting techniques. Section 5 provides some fundamentals and concepts useful to understand the Deep Learning models commonly used in the energy domain. Section 6 collects different experiences of using these models in the context of smart grids for forecasting purposes. Finally, Section 7 summarizes the main conclusions of this work.

## 2. The Importance of Demand Forecasting

In [7] the authors summarize the main requirements of smart grids as follows: flexible enough to meet users’ needs, able to manage uncertain events, accessible for all users, reliable enough to guarantee high-quality energy delivery to consumers, and innovative enough to manage energy efficiently.

With these requirements in mind, smart grids should aim to develop low-cost, easy-to-deploy technical solutions with distributed intelligence to operate efficiently in today’s increasingly complex scenarios. To upgrade a traditional electrical grid into a smart grid, intelligent and secure communication infrastructures are necessary [4].

According to the study presented in [8], forecasting can be applied in two main areas: grid control and demand response. In [9], the authors highlight that forecasting models are essential to provide optimal quality of the energy supply at the lowest cost. In addition, real-time information on users’ energy consumption patterns will enable more sophisticated and efficient forecasting models to be applied. Forecasting must also consider the need to manage constantly changing information. In [10], the authors highlight that, with the smart grid, demand response programs can make the grid more cost efficient and resilient.

The authors in [11] remark that there are important challenges in demand forecasting due to the uncertainties in the generation profile of distributed and renewable energy generation resources. In fact, increasing attention is being paid to load forecasting models, especially dealing with renewable energy sources (solar radiation, wind, etc.) [9].

The distributed generation paradigm facilitates the use of renewable energy sources that can be placed near consumption points. When using this paradigm, smart grids have multiple small plants that supply energy to their surroundings. Consequently, the dependence on the distribution and transmission grid is smaller [9]. However, this paradigm makes grid control even more uncertain, especially when the distributed generation sources are renewable and consequently have a random nature. Despite this difficulty, the share in energy production of variable renewable energy sources is expected to increase in the coming years [12].

Another key element are microgrids (see definition in Table 1) [13,14]. Based on this concept, and taking into consideration the intelligence deployed in buildings, new concepts have emerged including smart homes (see definition in Table 1) and smart buildings (see definition in Table 1). Buildings today are complex combinations of structures, systems, and technology. Technology is a great ally in optimizing resources and improving safety. Advances in building technologies are combining networked sensors and data recording in innovative ways [15]. Modern facilities can adjust heating, cooling, and lighting to maximize energy efficiency, providing also detailed reports of energy consumption. In these new smart environments (see definition in Table 1), sensors and smart devices are deployed to obtain enough information about the users’ energy consumption patterns. Once again, this requires forecasting models that must be applied to the specific variables of the scenario to be controlled.

Forecasting models will allow to consider variables (climatic, social, economic, habit-related, etc.) that can influence the accuracy of forecasts [9]. These authors remark that energy demand estimates in disaggregated scenarios, such as residential users in smart buildings, are more complex compared to energy demand estimates for an aggregated scenario, such as a country. Disaggregating the demand also facilitates the implementation of demand response, as different prices can be offered based on the criteria set by the power company.

The gradual integration of intelligence at the transmission, distribution and end-user levels of the electricity system aims to optimize energy production and distribution to adjust producers’ supply to consumers’ demand. Moreover, smart grids seek to improve fault detection algorithms [16]. Accurate demand forecasts are very useful for energy suppliers and other stakeholders in the energy market [17]. In fact, load forecasting has been one of the main problems faced by the power industry since the introduction of electric power [18].

## 3. Important Factors in Demand Forecasting

Electricity demand is affected by different variables or determinants. These variables include forecasting horizons, the level of load aggregation, weather conditions (humidity, temperature, wind speed, and cloudiness), socio-economic factors (industrial development, population growth, cost of electricity, etc.), customer type (residential, commercial, and industrial), and customer factors in relation to electricity consumption (characteristics of the consumer’s electrical equipment) (e.g., [19,20,21,22,23]).

To fully understand demand forecasting techniques and objectives, it is necessary to examine these determinants. In this section, the authors will focus on (1) period, (2) economic issues, (3) weather conditions, and (4) customer-related factors.

### 3.1. Period or Forecasting Horizon

The period commonly referred as forecasting horizon is probably one of the factors that has the greatest impact.

According to different authors (e.g., [17,24]), demand forecasting can be classified into three categories with respect to the forecasting horizon:

- Short-term (typically one hour to one week).
- Medium-term (typically one week to one year).
- Long-term (typically more than one year).

Factors affecting short-term demand forecasting usually do not last long, such as sudden changes of weather [22]. The quality of short-term demand forecasting is critical for electricity market players [20]. On the other hand, the influencing factors of medium-term demand forecasting often have a certain time duration, such as seasonal weather changes. Finally, the factors influencing long-term demand forecasting last for a long time, typically several forecast periods, e.g., changes in Gross Domestic Product (GDP) [22]. Indeed, economic factors have an important impact on long-term demand forecasting, but also on medium and short-term forecasting [25].

The authors of [26] identify the following categories in relation to the forecasting horizon:

- Very short-term (typically seconds or minutes to several hours).
- Short-term (typically hours to weeks).
- Medium-term and long-term (typically months to years).

According to these authors, very short-term demand forecasting models are generally used to control the flow. Short-term demand forecasting models are commonly used to match supply and demand. And, finally, medium-term and long-term demand forecasting models are typically used to plan asset utilities.

The authors in [27] showed that the load curve of grid stations is periodic, not only in the daily load curve, but also in the weekly, monthly, seasonal, and annual load curves. This periodicity makes it possible to forecast the load quite effectively.

Demand also reflects the daily lifestyle of the consumer [28]. Consumers’ daily demand patterns are based on their daily activities, including work, leisure and sleep hours. In addition, there are other demand variations patterns over time. For example, during holidays and weekends, demand in industries and offices is significantly lower than during weekdays due to a drastic decrease in activity. Finally, power demand also varies cyclically depending on the time of the year, day of the week, and time of day [22].

### 3.2. Socio-Economic Factors

Socio-economic factors, including industrial development, GDP, and the cost of electricity, also significantly affect the evolution of demand. Indeed, as mentioned in the previous section, economic factors considerably affect long-term demand forecasts, and also have an important impact on medium- and short-term forecasts.

For example, industrial development will undoubtedly increase energy consumption. The same will be true for population growth. This means that there is a positive correlation between industrial development, or population growth, and energy consumption.

GDP is an indicator that captures a country’s economic output. Countries with a higher GDP generate a greater quantity of goods and services and will consequently have a higher standard of living and lifestyle habits, which will stimulate energy demand.

Another economic factor to consider is cost, as it also affects demand. For example, when the price of electricity decreases, wasteful electricity consumption tends to increase [22].

The cost of electricity depends on different factors and is shaped in different ways. For example, in some countries such as Spain, there are two markets (regulated and free) for electricity. In the free market, the cost of electricity is established in the contract signed by the consumer. In contrast, in the regulated market, the price of electricity depends on supply and demand. The price is updated hourly and fluctuates. From the demand side, the more electricity is demanded, the more expensive it is. When less electricity is demanded, the cheaper it is. Normally, it is cheap to use electricity at dawn and expensive to do it when everyone else is using it (e.g., at dinner time).

But it is not only the demand that influences prices, but also the supply of energy. The reason is that variations in the price of electricity on the regulated market are caused by differences between demand and supply. Consequently, supply must consider the different ways of generating electricity, which have different costs. The cheapest is electricity generated by renewable energies such as solar, wind and hydroelectric. The price of nuclear energy is also low; however, in many countries (e.g., Spain), nuclear energy does not cover all energy needs. Thermal (coal), cogeneration, or combined cycle—whose main fuel is gas—tends to be more expensive. It is also important to remember that the main sources of renewable energy, such as hydroelectric or wind, depend on uncontrollable external factors. For example, sufficient rainfall is essential to produce hydroelectric power. However, there is no way to control the weather to make it favorable for producing electrical energy. Given the above, the price is determined by the price of a mix of different sources of power generation, from cheapest to most expensive, until the entire energy demand is met.

### 3.3. Weather Condition

There are different weather variables relevant for demand forecasting such as temperature, humidity, and wind speed.

The influence of weather conditions on demand forecasting has attracted the interest of many researchers. As an example, the authors in [29] proposed different models to forecast next day’s aggregated load using Artificial Neural Networks (ANNs), considering the most relevant weather variables—more specifically, mean temperature, relative humidity, and aggregate solar radiation—to analyze the influence of weather.

Some authors have studied the relationship between temperature and electricity consumption and claim that the correlation between temperature and the electricity load curve is positive, especially in summer (e.g., [25]).

Currently, heat waves have become more common around the world, as well as the possibility of extreme temperatures. In addition, heat waves are not only more frequent, but also more intense and longer lasting. Moreover, the nights are getting warmer, which is an added problem. The main effect of a heat wave is an increase in energy consumption as the consumer turns on the air conditioning more and for longer periods of time. Additionally, cooling systems must work harder as they must cope with higher temperatures.

During the summer, heat waves force the grid to be at maximum capacity. In fact, one of the ways in which a heat wave affects consumption is through the increased saturation of the electrical grid. While cold waves are counteracted with electricity, gas, wood, etc., heat waves can only be fought with electricity. In other words, the devices that consumers use for cooling are mainly powered by electricity. For this reason, heat waves generate more stress on power lines, as well as higher consumption.

It should be noted that, in colder countries, the increase in consumption during a heat wave is usually lower. This is because the installation of air conditioning systems is not as common as in warmer countries. However, these colder countries are facing heat waves that did not occur in previous years (before climate change) and this is causing them all type of problems, as they are less prepared. This situation is forcing these countries to make changes such as increasing the use of cooling systems.

On the other side, experience of the harshness of temperature increases with humidity, especially during the rainy season and summer. For this reason, electricity consumption increases during humid summer days. It is also important to note that in coastal areas, such as the Mediterranean area in Spain, electricity consumption tends to be higher. This is both because houses tend to have more electrical equipment than in other areas, and because of the high degree of humidity due to the proximity of the sea.

Wind speed also affects electricity consumption. When it is windy, the human body feels that the temperature is much lower and more heating is needed, which increases electricity consumption. However, it should also be noted that wind energy is one of the main renewable energies. In other words, when there is wind, electricity consumption increases, but at the same time its price decreases. This is because, as explained in the previous section, the price of the electricity is usually determined as a mix of the different energy sources, from cheapest energies (renewables, including wind, and nuclear) to the most expensive generation sources (thermal, combined cycle).

Temperature, humidity, and wind affect the use of electricity. Humidity and temperature are also the main weather variables used in electricity demand prediction systems to minimize operating costs. However, other factors, such as clouds, also play a role. For example, during the day, when clouds disrupt sunlight there is usually a drop in temperature and, consequently, higher electricity consumption.

### 3.4. Customer Factors

The type of customer (residential, commercial, and industrial), as well as other customer factors related to electricity consumption (characteristics of the consumer’s electrical equipment) can also affect demand. This is important because most energy companies have different types of customers (residential, commercial, and industrial consumers), who have equipment that varies in type and size. These different types of customers have different load curves, although there are some similarities between commercial and industrial customers.

Table 2 summarizes the main determinants affecting electricity demand described in this section.

## 4. Classification of Demand Forecasting Techniques

This section classifies demand forecasting models according to three different criteria: (1) period, (2) forecasting objective, and (3) type of model used.

The first classification focuses on the point of view of the period to be forecasted, i.e., the forecasting horizon. To select this criterion, the electricity demand determinants presented in the previous section have been considered. The second classification focuses on the point of view of the forecasting objective, differentiating between forecasting techniques that produce a single value and those that produce multiple values. Finally, the third classification focuses on the point of view of the model used.

### 4.1. Classification of Demand Forecasting Techniques according to the Forecasting Horizon

As explained in the previous section, the main forecasting horizons that can be identified are the following:

- Very short-term: typically from seconds or minutes to several hours.
- Short-Term: typically from hours to weeks.
- Medium-Term: typically from a week to a year.
- Long-Term: typically more than a year.

The main difference is the scope of the variables used in each case. Very short-term forecasting models use recent inputs (typically minutes or hours), short-term forecasting models use inputs typically in the range of days, and medium and long-term forecasting models use inputs typically in the range of weeks or even months.

Power companies are particularly interested in producing accurate forecasts for the load profile (e.g., [9,30,31]). This is because it can directly affect the optimal scheduling of power generation units. However, due to the non-linear and stochastic behavior of consumers, the load profile is complex, and although research has been done in this area, accurate forecasting models are still needed [32].

### 4.2. Classification of Demand Forecasting Techniques by Forecasting Objective

Forecasting models can be also classified according to the number of values to be forecasted. In this case, two main categories can be considered.

The first category refers to forecasting techniques that produce only one value (e.g., next day’s total load, next day’s peak load, next hour’s load, etc.). Examples are found in [33,34].

The second category refers to forecasting techniques that produce multiple values, e.g., the next hours’ peak load plus another parameter (e.g., the aggregate load) or the load profile. Examples are found in [35,36,37].

Generally speaking, one-value forecasts are useful for optimizing the performance of load flows. On the other hand, multiple-value forecasts are mainly used for energy generation scheduling [9].

### 4.3. Classification of Demand Forecasting Techniques according to the Model Used

The model to be used is usually decided by the practitioner. In terms of models, the main groups are linear and non-linear approaches.

Linear models include Spectral Decomposition (SD), Partial Least-Square (PLS), Auto-Regressive Integrated Moving Average (ARIMA), Auto-Regressive Conditional Heteroscedasticity (ARCH), Auto-Regressive (AR), Auto-Regressive and Moving Average (ARMA), Moving Average Model (MAM), Linear Regression (LR), and State-Space (SS).

Linear techniques have progressively lost importance and interest in favor of non-linear techniques based on ANNs. Deep Learning models use ANNs, inspired by the human nervous system. These models can learn patterns from the data generated and forecast peak demand in the context of today’s complex smart scenarios, where a large amount of data is continuously generated from different sources [7].

Table 3 summarizes the criteria commonly used to classify demand forecasting models.

## 5. Fundamentals and Concepts of Machine Learning and Deep Learning Systems

Artificial Intelligence is a complex concept that, in a nutshell, refers to machine intelligence [38]. Unlike humans, Artificial Intelligence can identify patterns within a large amount of data using a quite limited amount of time and resources. Furthermore, the computational capacity of machines does not decrease with time and/or fatigue [39].

Artificial Intelligence systems use different type of learning methods, such as Machine Learning and Deep Learning.

### 5.1. Machine Learning

Machine Learning algorithms are pre-trained to produce an outcome when confronted with a never-before-seen dataset or situation [40]. However, the computer needs more examples to learn than humans do [41]. Machine Learning allows the introduction of intelligent decision-making in many areas and applications where developing algorithms would be complex and excellent results are needed [42].

There are different categories of Machine Learning algorithms including supervised, semisupervised, unsupervised, and reinforcement learning. These different categories of algorithms are briefly described below.

#### 5.1.1. Supervised Learning

After being trained with a set of labelled data examples, these algorithms can predict label values when the input has unlabeled data. The problems typically associated with this type of learning are (1) regression and (2) classification [43].

In regression the algorithm focuses on understanding the relationship between dependent and independent variables. In classification, the algorithm is used to predict the class label of the data. Common classification problems include (1) binary classification, between two class labels; (2) multi-class classification, between more than two class labels; and (3) multi-label classification where one piece of data is associated with several classes or labels, as opposed to traditional classification problems with mutually exclusive class labels [44].

Methods used for supervised learning include Linear Discriminant Analysis (LDA), Naive Bayes (NB), K-nearest Neighbors (KNN), Support Vector Machine (SVM), Decision Tree (DT), Random Forest (RF), Adaptive Boosting (AdaBoost), Extreme Gradient Boosting (XGBoost), Stochastic Gradient Descent (SGD), Rule-based Classification (for classification); and LR, Non-Linear Regression (NLR), and Ordinary Least Squares Regression (OLS) (for regression) [44,45]. The most widely cited and implemented supervised learners in the literature are DT, NB, and SVM algorithms [46].

Some interesting practical applications are text classification, predicting the sentiment of a text (such as a Tweet or other social media), assessing the environmental sustainability of clothing products [47], characterizing, predicting, and treating mental disorders [48], and estimating peak energy demand.

#### 5.1.2. Unsupervised Learning

This type of learning uses unlabeled data. In this case, the system explores the unlabeled data to find hidden structures, rather than predicting the correct output. This type of learning is not directly applicable to regression or classification problems, as the possible values of the output are unknown [49]. Instead, it is often used for (1) clustering, (2) association, and (3) dimensionality reduction [43].

Clustering allows unlabeled data to be grouped based on their similarities or differences [49,50]. Association uses different rules to identify new and relevant insights between the objects of a set. Finally, dimension reduction allows a reduction of the number of features (or dimensions) of a dataset to eliminate irrelevant or less important features and thus reduce the complexity of the model [44]. This reduction in the number of features can be done by keeping a subset of the original features (feature selection) or by creating completely new features (feature extraction).

The most popular clustering algorithm is probably K-means clustering, where the *k* value represents the size of the cluster [44,45,51]. Association algorithms include Apriori, Equivalence Class Transformation (ECLAT), and Frequent Pattern (F-P) Growth algorithms. Finally, dimensionality reduction typically uses the Chi-squared test, Analysis of Variance (ANOVA) test, Pearson’s correlation coefficient, Recursive Feature Elimination (RFE) for feature selection, and Principal Components Analysis (PCA) for feature extraction.

According to [46], the most commonly used unsupervised learners are K-means, hierarchical clustering, and PCA.

These unsupervised learners can have many practical applications, such as facial recognition, customer classification, patient classification, detecting cyber-attacks or intrusions [52], and data analysis in the astronomical field [53].

#### 5.1.3. Semisupervised Learning

Conceptually situated between supervised and unsupervised learning, this type of learning allows the taking advantage of the large unlabeled datasets that are available in some cases combined with (usually smaller) amounts of labelled data [54,55]. This opens up interesting possibilities as labelled data are often scarce, while unlabeled data are more frequent, and a semisupervised learner can obtain better predictions than those produced using only labelled data [44].

Candidate applications are those where there is only a small set of labelled examples, and many more unlabeled ones, or when the labelling effort is too high. An example is medical imaging, where a small amount of training data can provide a large improvement in accuracy [43,56].

Table 4 compares Supervised and Unsupervised learning, focusing on the type of input data used in each case (labeled versus unlabeled data), and the main tasks for which both types of learning are used (classification, regression versus clustering, association, and dimensionality reduction).

#### 5.1.4. Reinforcement Learning

This learning technique depends on the relationship between an agent performing an activity and its environment, which provides positive or negative feedback [57,58]. The agent must choose actions that maximize the reward in that environment. Popular methods include Monte Carlo, Q-learning, and Deep Q-learning [44].

Traditionally common applications include strategy games such as chess, autonomous driving, supply chain logistics and manufacturing, genetic algorithms [57], 5G mobility management [59], and personalized care delivery [60].

### 5.2. Deep Learning

Machine Learning can be classified into shallow and deep, considering the complexity and structure of the algorithm [41]. Deep Learning uses multiple layers of neurons composed of complex structures to model high-level data abstractions [61]. The type of output and the characteristics of the data determine the algorithm to be used for a particular use case [62].

Deep Learning uses ANNs inspired by the human nervous system [63]. This type of network typically has two layers of input and output nodes respectively, connected to each other by one or more layers of hidden nodes. Possible deep ANN architectures include Multilayer Perceptron (MLP), Long Short-Term Memory Recurrent Neural Network (LSTM-RNN), Generative Adversarial Network (GAN), and Convolutional Neural Network (CNN or ConvNet).

According to our literature review, the most widely used models in the energy domain are Convolutional Neural Networks (CNNs), Recurrent Neural Networks (RNNs), Long Short-Term Memory (LSTM), Deep Q-Networks (DQNs) and Conditional Restricted Boltzmann Machine (CRBM) and a variation of any of them, a combination of two or more of them, or the combination of any of them with other techniques. These models are briefly described below.

#### 5.2.1. Convolutional Neural Networks

**Figure 1:**
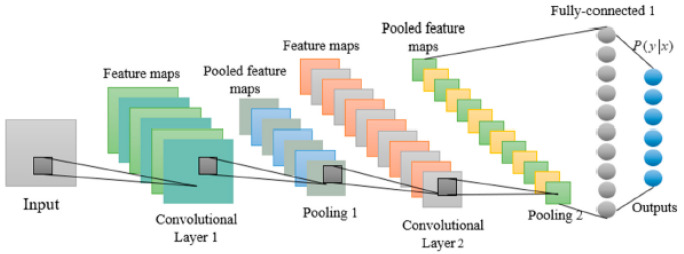
Standard Convolutional Neural Network Architecture [65].

These networks are biologically inspired networks, like the ordinary neural networks. However, in this type of network the inputs are assumed to have a specific structure such as images [64]. Being one of the most widely used and effective models for Deep Learning, these networks usually include two types of layers (i.e., pooling and convolution layers). A typical CNN architecture usually consists of an input layer, a convolutional layer, a Max pooling layer, and the final fully connected layer, as shown in Figure 1 [65].

The total input to the *j*th feature map of layer *l* at position (x,y) can be expressed [66]:

(1) $$v_{j}^{\left(l\right)}\left(x,y\right)=\overset{l}{\underset{i=1}{\sum }}\overset{F-1}{\underset{u,v=0}{\sum }}k_{ji}^{\left(l\right)}\left(u,v\right)\cdot O_{i}^{\left(l-1\right)}\left(x-u,\;y-v\right)+b_{j}^{\left(l\right)}\;$$

Convolutional layer output:

(2) $$O_{i}^{\left(l-1\right)}\left(x,y\right)=f\left(v_{j}^{\left(l\right)}\left(x,\;y\right)\right)\;$$

Pooling layer output:

(3) $$O_{i}^{\left(l+1\right)}\left(x,y\right)=\underset{u,v=0,\ldots ,G-1}{\max}O_{i}^{\left(l\right)}\left(x\cdot s+u\;,\;y\cdot s+v\right)\;$$

where $O_{i}^{\left(l-1\right)}\left(i=1,\ldots ,l\right)$ represents feature maps on the (*l + 1*) layer; $k_{ji}^{\left(l\right)}\left(u,v\right)$ denotes trainable convolution kernel; $b_{j}^{\left(l\right)}$ indicates trainable bias; *G* is pooling size; and *S* means stride.

Different architectural designs explore the effect of multilevel transformations on the learning ability of such networks. One of these possible architectural designs is Pyramid. The Pyramid Architecture of Convolutional Neural Network is commonly known as Pyramid-CNN [66].

#### 5.2.2. Recurrent Neural Networks

**Figure 2:**
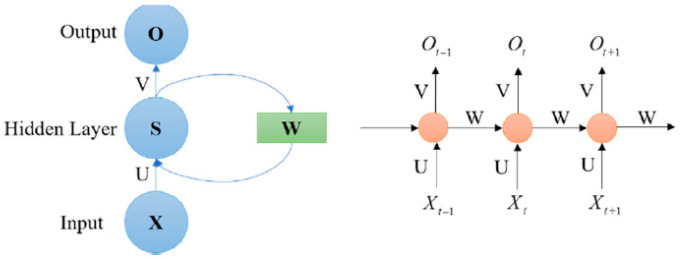
Framework of a Recurrent Neural Network: Input layer (*X_t_*); output layer (*O_t_*); hidden layer (*S_t_*); parameter matrices and vectors (*U, V, W*); activation function of output layer (*σ_y_*); and activation function of hidden layer (*σ_h_*) [65].

In this type of network, the connections between nodes form a directed or undirected graph along a time sequence. Figure 2 shows a typical RNN structure [65].

This network can use a gating mechanism called Gated Recurrent Units (GRUs) and introduced in 2014 by the authors in [67]. GRU are like LSTM networks but with a forgetting gate and fewer parameters as they lack an output gate.

Another variation of this type of network, proposed by Elman [68], is the Elman RNN that includes modifiable feedforward connections and fixed recurrent connections. It uses a set of context nodes to store internal states, which gives it certain unique dynamic characteristics over static ones [69].

#### 5.2.3. Long Short-Term Memory

These networks are a special kind of RNN. Unlike standard feedforward neural networks, these networks have feedback connections, and can even process entire sequences of data (such as speech or video), in addition to individual data points (such as images).

**Figure 3:**
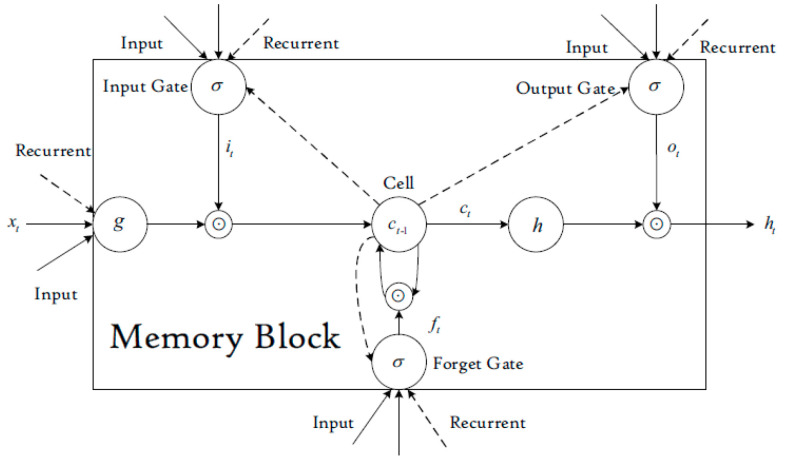
LSTM memory block [70].

This type of RNN contains an input layer, a recurrent hidden layer, and an output layer, with a memory block structure as shown in Figure 3 [70].

The LSTM memory block can be described according to the following equations [70]:

(4) $$i_{t}=\sigma (W_{i}x_{t}+U_{i}h_{t-1}+b_{i})$$

(5) $$f_{t}=\sigma \left(W_{f}x_{t}+U_{f}h_{t-1}+b_{f}\right)$$

(6) $$c_{t}=f_{t}⊙c_{t-1}+i_{t}⊙g\left(W_{c}x_{t}+U_{c}h_{t-1}+b_{c}\right)$$

(7) $$O_{t}=\sigma \left(W_{O}x_{t}+U_{O}h_{t-1}+V_{O}c_{t}+b_{O}\right)$$

(8) $$h_{t}=O_{t}⊙h\left(c_{t}\right)$$

where *x_t_* is the model input at time *t*; *W_i_*, *W_f_*, *W_c_*, *W_0_*, *U_i_*, *U_f_*, *U_c_*, *U_0_*, *V_0_* are weight matrices; *b_i_*, *b_f_*, *b_c_*, *b_0_* are bias vectors; *i_t_*, *f_t_*, *0_t_* are respectively the activations of the three gates at time *t*; *c_t_* is the state of memory cell at time *t*; *h_t_* is the output of the memory block at time *t*; ⊙ represents the scalar product of two vectors; σ(x) is the gate activation function; *g(x)* is the cell input activation function; *h(x)* is the cell output activation function.

A possible extension of this model is the Bidirectional LSTM (B-LSTM). The aim of this type of LSTM network is to analyze sequences from both front-to-back and back-to-front, i.e., the sequence information flows in both directions backwards and forwards, unlike in a normal LSTM.

#### 5.2.4. Deep Q Network and Dueling Deep-Q Network

**Figure 4:**
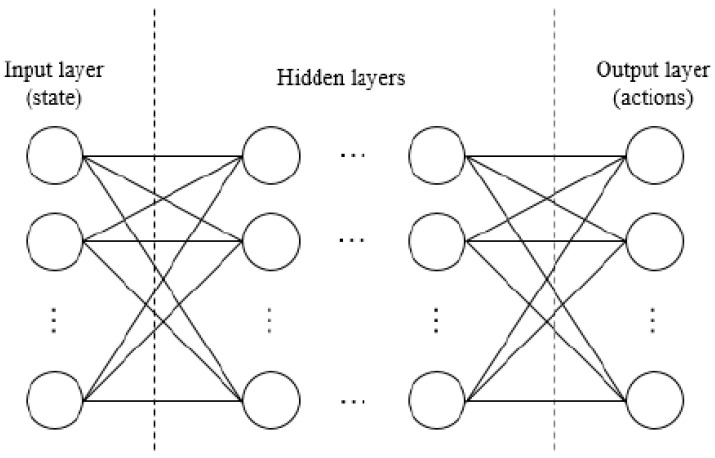
Deep Q-Network architecture [71].

Deep Q Network (DQN) and Dueling Deep-Q Network (DDQN) are a type of ANN using the Deep Q learning algorithm, which is popular in reinforcement learning. In a dueling network there are two streams to separately estimate the state-value as well as the advantages for each action. The main objective of Deep-Q Network is to choose the best action in a certain state. Considering *π* is the policy followed by an agent in a given environment, the function *Q_π_* can be defined as follows [71]:

(9) $$Q_{\pi }\left(s,\;a\right)=E\left[r_{1}+\gamma r_{2}+\ldots |S_{0}=s,A_{0}=a,\pi \right]\#\;$$

where *s* is a state; *a* is an action; *r_i_* is the potential reward; γ∈[0,1] is a discount factor for making the immediate reward more important than the futures ones. Therefore, the objective of Q-learning is to maximize the optimized value function Q^*^(s,a) = max _π_Q _π_(s,a). Figure 4 shows the scheme of a typical DQN architecture [71].

#### 5.2.5. Conditional Restricted Boltzmann Machine

A Restricted Boltzmann Machine (RBM) is a stochastic RNN with two layers, one with visible units and one with binary hidden units. This type of network can learn a probability distribution over its set of inputs. RBMs are a variant of Boltzmann Machines (BM) and can be in supervised or unsupervised mode, depending on the task to be performed.

When BMs are restricted, a pair of nodes from each of the two groups of units (visible and hidden) can have a symmetric connection between them, but there are no connections between nodes in the same group, allowing for more efficient training. On the other hand, unrestricted BMs can have connections between hidden units.

**Figure 5:**
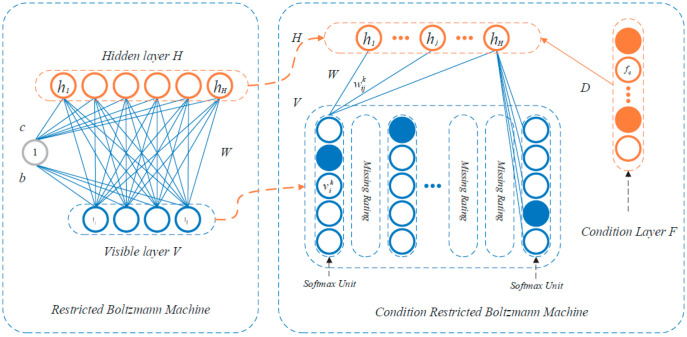
Restricted Boltzmann machine [73].

RBM consists of *m* visible units *V = (v*_1_,…, *v_m_)* representing observable data and *n* hidden units *H = (h*_1_,…, *h_n_)* capturing dependencies between observable variables, with the conditional layer units *F = (f*_1_,…, *f_p_)*, as it is shown in Figure 5 [72].

The energy function of CRBM is [73]:

(10) $$E\left(V,\;H,\;F\right)=-\sum_{i=1}^{m}\sum_{k=1}^{K}v_{i}^{k}b_{i}^{k}-\sum_{i=1}^{m}\sum_{j=1}^{H}\sum_{k=1}^{k}v_{i}^{k}W_{ij}^{k}h_{j}-\sum_{j=1}^{H}h_{j}b_{j}-\sum_{j=1}^{H}\sum_{q=1}^{F}f_{q}D_{q_{j}}\;$$

where *m* represents the number of items the user rated; *H* is the number of hidden layers; *F* is the number of conditional layers; *K* is the highly rating; $v_{i}^{k}$ is the binary value of visible layer unit *i* and rating *k*; *h_j_* is the binary value of hidden unit *j*; *f_q_* is the binary value of conditional layer *F*; $b_{i}^{k}$ is the bias of rating *k* with visible layer unit *i*; *b_j_* is the bias of feature *j*; $W_{ij}^{k}$ is the connected weight between hidden layer *H* and visible layer *V*; *D* is the connected weight between hidden layer H and conditional layer *F*; *D_qj_* is the connected weight between hidden feature j and conditional layer unit *q*.

In [73], the authors introduced the Factored Conditioned Restricted Boltzmann Machines (FCRBMs) by adding the concept of factored, multiplicative, and tridirectional interactions to predict multiple human movement styles.

Finally, Deep Belief Networks (DBNs) are formed by several RBMs stacked on top of the other [74].

## 6. Deep Learning Models and Demand Forecasting in the Context of Smart Grids

Researchers have proposed forecasting models in the two main areas where Deep Learning techniques can be applied [8]: (1) demand management (e.g., [75,76]) and (2) grid control (e.g., [77,78,79]).

Due to the growing demand for energy from different sectors, supply and demand must be balanced in the electrical grid. In this scenario, smart grids can play an important role by providing a bidirectional flow of energy between consumers and utilities. Unlike traditional electrical grids, smart grids have sophisticated sensing devices that generate data from which energy patterns can be derived. These patterns are extremely useful for load forecasting, peak shaving, and demand response management.

As the amount of data generated by a smart grid is huge and constantly increasing, Deep Learning based models are a good option to understand consumption patterns and make forecasts. Researchers have studied the possibilities of using Deep Learning models, with LSTM networks playing a leading role (e.g., [32,57,80]).

Table 5 summarizes different works where practitioners have successfully used Deep Learning techniques for forecasting purposes. These experiences have been identified after a systematic review of the published literature. The search for references has been carried out in different scientific databases (e.g., ScienceDirect, SpringeLink, IEEExplore, etc.). Papers from various relevant scientific journals such as Energy Informatics, IEEE Transactions on Smart Grid, Energies and Applied Energy have also been reviewed. The keywords used include:

- terms like Deep Learning, ANN, neural networks, and the names of different Deep Learning models, both full and acronyms (e.g., Long Short-Term Memory networks and LSTM),

combined (AND) with:

- terms related to the energy field, more specifically, “energy demand forecasting”, “electricity demand forecasting”, “load forecasting”, “demand response”, “demand-side response” and variations of these expressions.

The search was limited to the last 6 years. The decision as to which articles were finally included in Table 5 was made by the authors after reviewing the search results and ensuring that the work involved the use of a Deep Learning model for demand or load forecasting purposes.

## 7. Conclusions

Increasing energy demand puts pressure on the power grid to balance supply and demand. Smart grids can play an important role. In these systems, data related to energy use are regularly collected and analyzed to obtain energy consumption. The usage patterns obtained can be useful for demand and load forecasting. This is a challenging task in the context of smart environments, which is why researchers are putting special efforts into this.

To meet today’s demand forecasting challenges, where smart grids generate large amounts of data, it is necessary to use modern data-driven techniques. Deep Learning based models are a good alternative. Traditionally, research has focused on forecasting customers’ energy consumption using the small historical data sets available on their behavior. However, current research applying Deep Learning methods has demonstrated better performance than conventional forecasting methods. The use of Deep Learning models involves using large amounts of data, such as those provided by the different datasets used by practitioners in the works collected in Table 5. It is a fact that smart grids generate large amounts of data, so Big Data is also a key technology to overcome the challenges of renewable energy integration, load fluctuation and sustainable development. With the introduction of renewables into the smart grid, an increasing number of variables are brought into the system and more data need to be processed. This situation is also aggravated with the gradual introduction of electric vehicles, so these Big Data technologies are also becoming increasingly necessary [124].

The study conducted has revealed that the most widely used Deep Learning models in the energy domain for demand forecasting purposes are CNNs, RNNs, LSTM, DQNs, and CRBM and a variation of any of them, a combination of two or more of them, or the combination of any of them with other techniques. Notable are CNN and its variations such as Pyramid-CNN [82,85,88,90,91,94,95,101,106,107,109,115,118,119,123], LSTM and its variations such as B-LSTM [80,82,86,87,88,91,93,94,95,99,100,103,104,106,107,109,110,111,112,113,118,119,122], and a combination of both [82,88,91,94,95,106,107,109,118,119]. Real testbeds with high-quality data are not common, but are necessary to determine the performance of Deep Leaning models. It is important to continue testing future Deep Learning models, including potential variations and/or combinations of two or more models, for forecasting purposes in the context of smart grids. It is also important that these tests are carried out for different scenarios. Deep Learning models capable of automatically forecasting load for different types of customers, premises/buildings, and different weather conditions are still needed. It is important to test the performance of Deep Learning, but also to determine which model is best for each scenario.

In terms of datasets, practitioners used different options, highlighting PJM electricity market [32,92,102,108,112], SGSC [85,90,98], CER [98,114,120], ISO-NE [105,109,115], Pecan Street Inc. [80,97], UCI [106,107], UKDALE [113] and REDD [21]. Many reviews on demand/load forecasting in the context of smart grids focus on the Deep Learning models used but forget about the data. However, for a Deep Learning implementation to be successful, the algorithms are as valuable as the data. In fact, it would be desirable for researchers to incorporate more information about the data used in their works, addressing for example the training/validation/testing data split, the sampling interval of the data, the method for data cleaning, etc. One of the limitations of using Deep Learning models is the lack of high-quality real-world datasets. A future trend would probably be to shift the emphasis from the model to the data. Furthermore, the authors foresee an integration of IoT into Deep Learning models used for demand/load forecasting. IoT is enabling the democratization of sensing. This opens exciting opportunities in terms of high-quality data collection, which is critical in the context of demand/load forecasting. Related to this, another future trend would be the development of integrated systems that include the necessary data acquisition and pre-processing.

Finally, it is also remarkable that in most cases researchers focused on short-term forecasting.

Load forecasting is a challenging task in the context of smart environments. Consequently, researchers are putting special efforts into it. Real testbeds with high-quality data are not common but necessary to determine the performance of the Deep Leaning models. Deep Learning models capable of automatically forecasting load for different types of customers, premises/buildings, and different weather conditions are still needed.

## Figures and Tables

**Table 1:** Important keywords for reading this article.

Keyword	Definition
Demand forecasting	The process of estimating end-user demand in relation to electricity consumption over a given period.
Demand response	A change in end-users’ electricity consumption to better balance power demand and supply. Also referred to as demand-side response.
Load forecasting	The technique used by power companies to forecast the electricity needed to meet demand to better balance power demand and supply.
Load profile	The electricity demand forecast made for the next 24 h.
Microgrid	An aggregation of loads and microsources operating as a single system, i.e., a small electrical grid of users with local control and supply capability. It is normally connected to a centralised national electricity grid but can operate autonomously.
Traditional electrical grid	A grid developed specifically for the transmission of electrical energy from points of production to the utilization points. Also referred to as conventional/existing electrical/power grid.
Smart building	A building that uses a wide range of technologies to enable efficient use of resources, including electrical energy, while providing a comfortable environment for its occupants.
Smart environment	Any type of environment, such as a smart home or a smart building, that uses a wide range of technologies to enable efficient use of resources, including electricity, while creating a comfortable environment for its occupants.
Smart grid	An electrical grid that can monitor and provide real-time information on its own activity with the help of digital technologies. It is considered a developed form of the traditional electrical grid.
Smart home	A home that uses a wide range of technologies to enable efficient use of resources, including electricity, while creating a comfortable environment for its occupants.

**Table 2:** Main variables affecting electricity demand.

Determinant	Refers to…
Forecasting horizon	The time horizon for which demand forecasts are prepared.
Socio-economic factors	Industrial development, population growth, cost of electricity, and any other socio-economic factors that may influence end-users’ demand.
Weather conditions	Temperature, humidity, wind speed, and any other weather conditions that may influence end-users’ demand.
Customer factors	Type of customer (residential, commercial, and industrial), characteristics of the consumer’s equipment, and any other customer factors that may affect the end-users’ demand.

**Table 3:** Main criteria commonly used to classify demand forecasting models.

Criteria	Refers to…
Forecasting horizon	The time horizon for which electricity demand forecasts are prepared. The main forecasting horizons are:
	- very short-term: from seconds or minutes to several hours. - short-term: from hours to weeks. - medium-term: from a week to one year. - long-term: more than one year.
Aim of prediction	The number of values to be forecasted, mainly one value (e.g., next day’s total load, next day’s peak load, next hour’s load, etc.) or multiple values (e.g., the load profile).
Type of model used	Mainly linear (e.g., SD, PLS, ARIMA, ARCH, AR, ARMA, MAM, LR, SS, etc.), and non-linear ANN-based models.

**Table 4:** Supervised vs unsupervised learning.

Supervised Learning	Unsupervised Learning
It refers to:	
Developing predictive models based on input and output data	Grouping and interpreting data based on input data
Uses as input:	
Labelled data	Unlabelled data
Typically used for:	
Classification Regression	Clustering Association Dimensionality reduction

**Table 5:** Examples of application of Deep Learning techniques in the energy domain, focusing on demand/load forecasting.

Reference	Year	Models	Forecasting Horizon	Dataset	Outcome
Rodríguez et al. [81]	2022	Feedforward Spatiotemporal Neural Network + t-Student PDF (Probability Distribution Function)	Minutes	Solar irradiation database of the meteorological stations of Vitoria-Gasteiz, Spain, provided by the Meteorological Agency of the Basque Government: Euskalmet (http://www.euskalmet.euskadi.eus/) (accessed on 26 November 2022).	Solar irradiation forecaster satisfying the required condition Prediction Interval Coverage Percentage (PICP)_0.05_ > 0.95 on 85.22% of the days in 2017.
Taleb et al. [82]	2022	CNN + LSTM + MLP	Half-hourly 1-day 1-week	Data from EDM (Électricité de Mayotte) Open Data (https://opendata.electricitedemayotte.com/) (accessed on 26 November 2022).	Demand forecasting with Mean Absolute Percentage Error (MAPE) of 1.71% for 30-min predictions, 3.5% for 1-day predictions, and 5.1% for 1-week predictions.
Xu et al. [83]	2022	RBM	1-day 1-year	Historical load data on similar days from past years. Data from March 2010 to March 2018 were used for training and from March 2019 to March 2020 for validation.	Demand forecasting with mean of MAPE of less than 5% for all days in 1-day forecasts and MAPE less than 5% for 1-year forecasts.
Yem Souhe et al. [84]	2022	Support Vector Regression (SVR) + Firefly Algorithm (FA) + Adaptive Neuro-Fuzzy Inference System (ANFIS)	Years	Smart meter consumption data over a 24-year period (1994 to 2017) in Cameroon, obtained from the Electricity Distribution Agency, the Electricity Sector Regulatory Agency of Cameroon, and the World Bank.	Demand forecasting with Root Mean Square Error (RMSE) of 0.1524, Mean Absolute Error (MAE) of 21.023, and MAPE of 0.4124% showed that the proposed method outperformed other models such as LSTM and RF.
Aurangzeb et al. [85]	2021	Pyramid-CNN	Half-hourly	Data from individual customers who have a hot water system installed, from the Australian Government’s Smart Grid Smart City (SGSC) project database, launched in 2010, which contains data on thousands of individual household energy customers.	Demand forecasting for randomly selected customers from low and high consumption customer groups with an improvement of MAPE up to 10% compared to LSTM.
Jahangir et al. [86]	2021	B-LSTM	Hourly	Dataset for the province of Ontario (Canada). Hourly electricity price, load demand, and wind speed data for 3 years (from 1 January 2016, to 30 December 2018, with 1-h time intervals).	Improved forecasting results for wind speed, load, and electricity price, especially at peak points, based on RMSE, MAE, and MAPE, compared to other models such as LSTM and CNN.
Mubashar et al. [87]	2021	LSTM	1-month	Data from 12 houses over a period of 3 consecutive months.	Comparison of LSTM residential load demand forecasting with ARIMA and Exponential Smoothing, for individual and aggregate residential load demand, using MAE.
Rosato et al. [88]	2021	CNN + LSTM	1-day 3-days 7-days	Data from the NWTC photovoltaic power production plant, geographically identified by coordinates: 3954038.200 N, 10514004.900 W, elevation 1855 m, located in Denver, Colorado, United States. Irradiance data, along with other meteorological factors, were retrieved through the Measurement and Instrumentation Data Centre (MIDC) database. All time series were collected in the same plant, sampled hourly (i.e., 24 samples a day) and referred to the years 2017 and 2018.	Experimental results for output power forecasting, based on RMSE, showed that the proposed 2D-CNN model outperformed a multivariate implementation of LSTM.
Zhang et al. [89]	2021	DQN	3-h	Smart thermostats’ data collected during 1 month from a real building.	Automated building demand response control framework that enables cost-effective large-scale deployments. Cost analysis of a commercial building showed that the annual cost of optimal policy training was only 2.25% of the demand response incentive received.
Aurangzeb & Alhussein [90]	2020	Pyramid-CNN	Short-term (several time horizons)	Australian Government SGSC project database, initiated in 2010, containing data from thousands of individual household energy customers.	Accurate short-term demand forecasting of individual household customers with irregular energy consumption patterns. The results indicated that demand forecasting of individual households is highly unpredictable: more than 57% of the customers had more than 20 outliers in the daily energy consumption, yet the model fairly reasonably tracked the unpredictable energy consumption.
Bedi et al. [75]	2020	Elman RNN	1-day	Clemson University’s Real-Time Power and Intelligent Systems (RTPIS) Laboratory, South Carolina, United States.	Development of a smart building case study powered by the Internet of Things.
Escobar et al. [91]	2020	LSTM, CNN, GRU, and hybrid models: CNN-LSTM and CNN-GRU	3-days	4 years of hourly data from Madrid, including energy consumption, energy generation, pricing data, and meteorological information: temperature (K), humidity (water percentage in the air), wind direction in sexagesimal degrees, wind speed in miles per second (m/s), onshore wind and solar energy, and total load, these last in MW.	Comparative analysis of energy demand, and solar and onshore wind generation forecasting, for LSTM, CNN, GRU, and hybrid models merging CNN with LSTM and GRU, based on MAE, MAPE and RMSE. The combination of the best CNN and GRU models obtained better prediction results.
Hafeez et al. [32]	2020	FCRBM	Hourly	PJM electricity market. FE, Dayton, and EKPC power grids, United States.	Model compared to other forecasting methods such as LSTM.
Hafeez et al. [92]	2020	FCRBM	1-month	PJM electricity market, years 2014–2017, FE power grid, United States.	Hybrid electricity consumption forecasting model to provide efficient and accurate forecasting with an affordable convergence rate.
Hong et al. [21]	2020	Iterative Resblocks Based Deep Neural Network (IRBDNN)	1-week	REDD (Reference Energy Disaggregation Dataset), a publicly available dataset that records household appliance consumption data for residential users from March 2011 to July 2011.	IRBDNN model compared to existing methods such as DNN, ARMA and ELM, based on MAPE, RMSE and MAE, for residential buildings.
Nguyen et al. [93]	2020	LSTM	1-day	Electricity load consumption from 2012 to 2017 including about 2200 values in kWh, from Tien Giang, Vietnam.	Demand forecasting results evaluated using RMSE.
Rosato et al. [94]	2020	CNN + LSTM	3-days	Photovoltaic power production plant at Oak Ridge National Laboratory located in Oak Ridge, Tennessee, United States, with geographic coordinates: 3592099:600 N, 8430095:200 W, elevation 245 m. Irradiance data were retrieved through the MIDC database	Experimental results of load forecasting, based on RMSE, showed that the combination of CNN and LSTM outperformed the isolated use of LSTM.
Qi et al. [95]	2020	CNN + LSTM	1-day	Data from the integrated energy system of an industrial area in China, which is a combined electric, cooling, and heating system.	Experimental results showed that the CNN-LSTM composite forecasting model for short-term demand of individual household customers has a higher prediction accuracy than the CNN and LSTM models.
Wang et al. [96]	2020	Deep Reinforcement Learning (DRL) + DDQN	Hourly	Data from IEEE 33-node extension system (selected as a typical model of medium voltage distribution system model).	DDQN improves the noise and instability in traditional DQN, and reduces operation costs and peak load demand while regulating voltage to the safe limit.
Wen et al. [80]	2020	Deep Recurrent Neural Network (DRNN) + GRU + LSTM	Hourly	Dataport, Pecan Street Inc. Residential buildings, Austin, Texas, United States.	Deep learning model to forecast and fill in missing data on residential buildings energy demand.
Wen et al. [97]	2020	Modified RNN	Hourly	Dataport, Pecan Street Inc. Residential buildings, Europe.	Experimental results on residential buildings demand showed that peak demand can be reduced by 17%.
Yang et al. [98]	2020	Multitask Bayesian Neural Network (MT-BNN)	Hourly	Two public datasets on smart meters provided by the Irish Commission for Energy Regulation (CER) and the Australian Government’s SGSC project, respectively. The CER dataset was collected between July 2009 and December 2010 with the participation of more than 4225 residential customers and 2210 Small and Medium Enterprises (SMEs) participating. The SGSC dataset was collected for about 10,000 customers between 2010 and 2014 in New South Wales. Electricity consumption (kWh) was recorded every half hour at each meter in both datasets.	Experimental results, based on MAE and RMSE, showed that the proposed load forecasting framework for residential demand response provided higher accuracy of individual electricity consumption than other methods such as SVR, Gradient Boosted Regression Trees (GBRT), RF, and Pooling-based Long Short-Term Memory (PLSTM).
Amin et al. [99]	2019	LSTM	Several time horizons	Smart meter data collected over 2 years from 114 apartments, along with weather information for the same period.	Comparison of three demand forecasting methods: a piecewise LR model, the univariate seasonal ARIMA model, and a multivariate LSTM model. The results showed that while the LR model could be used for long-term planning, the LSTM model significantly improved the accuracy of short-term (1-day) demand forecasting compared to the ARIMA and LR models.
Atef & Eltawil [100]	2019	LSTM	Hourly	Real time electricity prices from Denmark from 17 January 2013 to 30 September 2018.	Comparative study of LSTM and SVR for electricity price forecasting in smart grids. Results showed that both models are effective. However, LSTM outperformed SVR, with a mean RMSE value of 1.1165 and 0.416 respectively.
Chan et al. [101]	2019	CNN	Short-term	Multivariate series composed of 9 variables and 2,075,259 observations provided by the Data Science and Interaction Scientific Team, Region de Paris, France. Data collected from December 2006 to November 2010 with a sampling frequency of 1 min.	Experimental results, based on MAE and RMSE, showed that the proposed CNN-based method achieves higher performance than the SVM model in demand forecasting with 0.514% MAE versus 14.32%, and 0.698% RMSE versus 19.23%.
Hafeez et al. [102]	2019	Modified Mutual Information (MMI) technique + FCRBM + Genetic Wind Driven Optimization (GWDO) algorithm	1-day	PJM electricity market	Experimental results showed that the proposed fast and accurate model outperformed existing models such as Multiple Instance Artificial Neural Network (MI-ANN) and Accurate Fast Converging Short-Term Load forecast (AFC-STLF), in terms of demand forecasting accuracy and convergence rate. The forecasting accuracy was improved using the MMI technique and the FCRBM model, and the convergence rate was enhanced with the GWDO algorithm.
Kaur et al. [103]	2019	RNN + LSTM	Hourly	Smart meter data of energy consumption in kWh from 112 households for 500 days with a sampling frequency of half an hour.	Experimental results, based on MAPE and RMSE, showed that the proposed method gives better results for smart homes demand forecasting than RNN and ARIMA.
Khafaf et al. [104]	2019	LSTM	Hourly 3-days 15-days	Historical energy consumption. For 3-day forecasts, the data corresponds to daily consumption for each month. For 15-day forecasts, the data corresponds to daily consumption for the whole year.	Experimental results, based on MAPE and RMSE, showed a good performance of the proposed LSTM model for demand forecasting.
Khan et al. [105]	2019	Combine Feature Selection Convolutional Neural Network (CFSCNN)	Half hourly 1-day 1-week 1-month	Market data from the ISO New England Control Area (ISO NE-CA) from January 2017 to December 2017 with half-hourly sampling frequency.	Experimental results, based on MAE and MSE, showed better efficiency and accuracy of the proposed model for demand forecasting compared to DB-SVM (Density Based Support Vector Machine).
Kim & Cho [106]	2019	CNN + LSTM	Minutely Hourly Daily Weekly	Individual household electricity consumption in the UCI Machine Learning repository of the University of California, Irvine, which provides a dataset of electricity consumption with 2,075,259 time-series and 12 variables (https://archive.ics.uci.edu/mL/datasets/) (accessed on 26 November 2022). The dataset collected electricity consumption over 4 years (from 16 December 2006 to 26 November 2010) in a household in France.	Experimental results for smart home demand forecasting, based on MSE, RMSE, MAE, and MAPE showed that the proposed model outperformed other techniques such as LSTM, GRU, B-LSTM, Attention-based LSTM, LR, FCRBM, and CRBM.
Kim & Cho [107]	2019	Particle Swarm Optimization (PSO) based CNN + LSTM	Minutely Hourly Daily Weekly	Individual household electricity consumption in the UCI Machine Learning repository of the University of California, Irvine, which provides a dataset of electricity consumption with 2,075,259 time series and 12 variables (https://archive.ics.uci.edu/mL/datasets/) (accessed on 26 November 2022). The dataset collected electricity consumption over 4 years (from 16 December 2006 to 26 November 2010) in a household in France.	Experimental results for smart home demand forecasting, based on MAE and MAPE, showed a better performance of the proposed model, which outperformed other techniques such as LR, RF, Regression, MLP, and CNN + LSTM (without PSO).
Lu & Hong [108]	2019	Reinforcement Learning (RL) + Deep Neural Network (DNN)	Hourly	PJM electricity market. Data from 1 January 2017 to 21 February 2018 were used to train the model.	Experimental results for demand forecasting and electricity prices, based on MAE and MAPE, showed good performance of the proposed model for service providers in purchasing energy from its various customers to improve grid reliability and balance energy fluctuations.
Pramono et al. [109]	2019	CNN + LSTM	Hourly	Two different datasets: ENTSO-E (European Network of Transmission System Operators for Electricity) dataset, and ISO-NE (Independent System Operator New England) dataset.	Experimental results for demand forecasting, based on MAE, MAPE, and RMSE, showed better performance of the proposed model compared to other techniques.
Rahman et al. [110]	2019	RNN + LSTM	Daily Monthly Yearly	Dataset containing household electricity consumption data at a sampling frequency of 1 min from 2006 to 2010. The electricity consumption values were collected for different electrical appliances in the household.	Comparison of ARIMA and RNN with LSTM, Univariate LR, and Multivariate LR. Experimental results for demand forecasting in smart homes showed that all models could capture the general trend of the data, but exhibited different predictive capabilities. Best forecasting results were obtained with a joint method based on Mahalanobis distance.
Syed et al. [111]	2019	Recurrent Neural Network (RNN) + LSTM	1-day	Dataset containing energy consumption records for 5567 households from the UK Power Network’s Low Carbon London project from November 2011 to February 2014.	Experimental results for demand forecasting versus temperature, humidity, dew point, wind speed and UV Index, based on RMSE, showed better performance of the proposed Averaging Regression Ensembles model based on the LR and LSTM RNN ensemble compared to other techniques such as LR Model and Elaboration Likelihood Model (ELM).
Ustundag et al. [112]	2019	LSTM	Hourly	PJM Data Miner 2, 1–11 March 2019 (hourly load) New Jersey, United States.	The work showed that data privacy assurance can be obtained to varying degrees with tolerable degradation in load forecasting results.
Vesa et al. [113]	2019	MLP + LSTM	1-day intraday (4 h)	UK Domestic Appliance-Level Electricity (UKDALE) open-access dataset containing historical electricity consumption readings from 5 houses over 655 days taken at a sampling rate of 6 s.	Experimental results for individual and aggregated load forecasting in residential buildings, obtaining MAE—5.60 kWh, MAPE—1.59%, and RMSE—6.19 kWh, showed high accuracy of the proposed combined model for residential building energy demand forecasting. The combined model outperformed MLP and LSTM models.
Yang et al. [114]	2019	GRU	Hourly	Real-world smart meter dataset provided by the Irish CER. The study used data from 800 residents and 400 SMEs with a sampling frequency of half an hour from 1 August 2010 to 31 October 2010.	Experimental results for load forecasting, based on two typical probabilistic scoring methods (pinball loss score, and winkler score), showed better performance of the proposed model compared to other techniques such as Quantile Regression Forest (QRF), Quantile Regression Gradient Boosting (QRGB), and Quantile Long Short-Term Memory (QLSTM) Neural Network.
Zahid et al. [115]	2019	CNN	Hourly	ISO-NE dataset with 2018 load data.	Improved classifiers were used to forecast load and electricity prices.
Ouyang et al. [116]	2019	DBN	Hourly	One year grid load data collected in an urbanized area in Texas, United States.	Prediction accuracy of the proposed model was evaluated with MAPE, RMSE, and Hit Rate (HR). Experimental results for load forecasting, examined in four seasons independently, showed a higher prediction accuracy of the proposed Gumbel-Houggard Copula-DBN model, compared to other techniques such as SVR and DBN.
Hafeez et al. [117]	2018	FCRBM + CRBM	1-week with hourly resolution, in the middle of each season	Publicly available Kaggle repository from the 2012 global energy forecasting competition. The dataset consisted of hourly load (kW) from 20 United States utility zones and temperature from 11 stations from 1 January 2004 to 30 June 2008.	Experimental results for load forecasting, based on MAPE, NRMSE and correlation coefficient, showed that the proposed models were accurate and robust compared to ANN and CNN. The adopted stacked FCRBM achieved 99.62% accuracy with affordable runtime and complexity.
Koprinska et al. [118]	2018	CNN + LSTM	1-day	Electricity load datasets from Australia, Portugal, and Spain for two years, 2010 and 2011 (from 1 January 2010 to 31 December 2011), with hourly sampled data. The Australian data was from New South Wales, provided by the Australian Energy Market Operator. The Portuguese and Spanish data were provided by the Spanish Energy Market Operator.	Comparison of the performance of CNN with MLP and LSTM RNN for photovoltaic solar power and load forecasting. Experimental results showed that CNN and MLP had similar accuracy and training time, and outperformed the other models.
Kuo & Huang [119]	2018	CNN + LSTM	Next 3 days	United States District public consumption and electric load dataset from 2016 provided by the Electric Reliability Council of Texas.	The load forecasting performance of the proposed CNN-based method is compared with other techniques such as SVM, RF, DT, MLP and LSTM. Experimental results, based on MAPE and Cumulative Variation of Root Mean Square Error (CV-RMSE), showed very high forecasting accuracy for the proposed model.
Shi et al. [120]	2018	Pooling-based DRNN	Hourly	Data from the Smart Metering Electricity Customer Behaviour Trials (CBTs) initiated by the Irish CER, from 1 July 2009 to 31 December 2010, involving over 5000 Irish residential and SME consumers. Samples of half-hourly electricity consumption (kWh) were available for each participant, as well as customer type and tariff.	Experimental results for smart home load forecasting, based on RMSE, showed that the proposed model outperformed other techniques such as ARIMA by 19.5%, SVR by 13.1%, and classical deep RNN by 6.5%.
Ghaderi et al. [121]	2017	RNN	Hourly	Hourly wind speed data from Meteorological Terminal Aviation Routine (METAR) weather reports from 57 stations on the East Coast of the United States including stations in New York, Massachusetts, New Hampshire, and Connecticut. A time period from 6 January 2014 to 20 February 2014, with the most unstable wind conditions of the entire year, was considered as test set.	Experimental results for wind speed forecasting, based on MAE, RMSE and NRMSE, showed that the proposed model outperformed other techniques such as Persistence Forecasting, AR of order 1, AR of order 3 and Wavelet Transform-Artificial Neural Network (WT-ANN).
Jarábek et al. [122]	2017	LSTM		Dataset on electricity consumption of Slovak companies, collected in the framework of the project International Centre of Excellence for Research of Intelligent and Secure Information-Communication Technologies and Systems, from 1 July 2013 to 16 February 2015 with a sampling frequency of 15 min. Consumption data from 11,281 enterprises were aggregated into 1152 time series considering the enterprise’s postcode.	Research on the need for clustering when using LSTM with Sequence to Sequence (S2S) architecture for grid level load forecasting on real world data. A method without clustering (simple aggregation of the consumers) was compared with a method using k-shape clustering. Experimental results for aggregated load forecasting showed that more accurate predictions were obtained using k-shape clustering.
Li et al. [123]	2017	CNN	Minutes	Electricity load, from January 2014 to June 2016 in a large city in China.	Proposed method considering all external factors that influence load forecasting such as humidity, temperature, wind speed, etc.

## Data Availability

Not applicable.

## Abbreviations

The following abbreviations are used in this manuscript:

AdaBoost	Adaptive Boosting
AFC-STLF	Accurate Fast Converging Short-Term Load forecast
ANFIS	Adaptive Neuro-Fuzzy Inference System
ANN	Artificial Neural Network
ANOVA	Analysis of Variance
AR	Auto-Regressive
ARCH	Auto-Regressive Conditional Heteroscedasticity
ARIMA	Auto-Regressive Integrated Moving Average
ARMA	Auto-Regressive and Moving Average
B-LSTM	Bidirectional Long Short-Term Memory
BM	Boltzmann Machines
CBT	Customer Behavior Trial
CER	Commission for Energy Regulation
CFSCNN	Combine Feature Selection Convolutional Neural Network
CNN	Convolutional Neural Network
CRBM	Conditional Restricted Boltzmann Machine
CV-RMSE	Cumulative Variation of Root Mean Square Error
DB-SVM	Density Based Support Vector Machine
DBN	Deep Belief Network
DDQN	Dueling Deep-Q Network
DNN	Deep Neural Network
DQN	Deep Q-Network
DRL	Deep Reinforcement Learning
DRNN	Deep Recurrent Neural Network
DT	Decision Tree
ECLAT	Equivalence Class Transformation
EDM	Électricité de Mayotte
ELM	Elaboration Likelihood Model
Elman RNN	Elman Recurrent Neural Network
ENTSO-E	European Network of Transmission System Operators for Electricity
FA	Firefly Algorithm
FCRBM	Factored Conditioned Restricted Boltzmann Machine
F-P	Frequent Pattern
GAN	Generative Adversarial Network
GBRT	Gradient Boosted Regression Trees
GDP	Gross Domestic Product
GRU	Gated Recurrent Units
GWDO	Genetic Wind Driven Optimization
HR	Hit Rate
IoT	Internet of Things
IRBDNN	Iterative Resblocks Based Deep Neural Network
ISO-NE	Independent System Operator New England
ISO NECA	ISO New England Control Area
KNN	K-nearest Neighbors
LDA	Linear Discriminant Analysis
LR	Linear Regression
LSTM	Long Short-Term Memory
LSTM-RNN	Long Short-Term Memory Recurrent Neural Network
MAE	Mean Absolute Error
MAM	Moving Average Model
MAPE	Mean Absolute Percentage Error
METAR	Meteorological Terminal Aviation Routine
MI-ANN	Multiple Instance Artificial Neural Network
MIDC	Measurement and Instrumentation Data Centre
MLP	Multilayer Perceptron
MMI	Modified Mutual Information
MT-BNN	Multitask Bayesian Neural Network
NB	Naive Bayes
NLR	Non-Linear Regression
NRMSE	Normalized Root Mean Squared Error
OLS	Ordinary Least Squares Regression
PCA	Principal Components Analysis
PDF	Probability Distribution Function
PICP	Prediction Interval Coverage Percentage
PLS	Partial Least-Square
PLSTM	Pooling-based Long Short-Term Memory
PSO	Particle Swarm Optimization
Pyramid-CNN	Pyramid Convolutional Neural Network
QLSTM	Quantile Long Short-Term Memory
QRF	Quantile Regression Forest
QRGB	Quantile Regression Gradient Boosting
RBM	Restricted Boltzmann Machine
REDD	Reference Energy Disaggregation Data Set
RF	Random Forest
RFE	Recursive Feature Elimination
RL	Reinforcement Learning
RMSE	Root Mean Squared Error
RNN	Recurrent Neural Network
RTPIS	Real-Time Power and Intelligent Systems
S2S	Sequence to Sequence
SD	Spectral Decomposition
SGD	Stochastic Gradient Descent
SGSC	Smart Grid Smart City
SME	Small and Medium Enterprise
SS	State-Space
SVM	Support Vector Machine
SVR	Support Vector Regression
UKDALE	UK Domestic Appliance-Level Electricity
WT-ANN	Wavelet Transform-Artificial Neural Network
XGBoost	Extreme Gradient Boosting

## References

[1] Butt O.M., Zulqarnain M., Butt T.M. (2021). Recent advancement in smart grid technology: Future prospects in the electrical power network. Ain Shams Eng. J..

[2] Cecati C., Mokryani G., Piccolo A., Siano P. An Overview on the Smart Grid Concept. Proceedings of the IECON 2010—36th Annual Conference on IEEE Industrial Electronics Society.

[3] Vakulenko I., Saher L., Lyulyov O., Pimonenko T. A Systematic Literature Review of Smart Grids. Proceedings of the 1st Conference on Traditional and Renewable Energy Sources: Perspectives and Paradigms for the 21st Century (TRESP 2021).

[4] Yan Y., Qian Y., Sharif H., Tipper D. (2012). A survey on cyber security for smart grid communications. IEEE Commun. Surv. Tutor..

[5] Shawkat Ali A.B.M., Azad S.A. (2013). Demand forecasting in smart grid. Green Energy Technol..

[6] Prabadevi B., Pham Q.-V., Liyanage M., Deepa N., Vvss M., Reddy S., Madikunta P.K.R., Khare N., Gadekallu T.R., Hwang W.H. (2021). Deep learning for intelligent demand response and smart grids: A comprehensive survey. arXiv.

[7] Ferreira H.C., Lampe L., Newbury J., Swart T.G. (2010). Power Line Communications. Theory and Applications for Narrowband and Broadband Communications Over Power Lines.

[8] Vanting N.B., Ma Z., Jørgensen B.N. (2021). A scoping review of deep neural networks for electric load forecasting. Energy Inform..

[9] Hernández L., Baladrón C., Aguiar J.M., Carro B., Sánchez-Esguevillas A.J., Lloret J., Massana J. (2014). A survey on electric power demand forecasting: Future trends in smart grids, microgrids and smart buildings. IEEE Commun. Surv. Tutor..

[10] Javed F., Arshad N., Wallin F., Vassileva I., Dahlquist E. (2012). Forecasting for demand response in smart grids: An analysis on use of anthropologic and structural data and short-term multiple loads forecasting. Appl. Energy.

[11] Khodayar M.E., Wu H. (2015). Demand forecasting in the smart grid paradigm: Features and challenges. Electr. J..

[12] Boza P., Evgeniou T. (2021). Artificial intelligence to support the integration of variable renewable energy sources to the power system. Appl. Energy.

[13] Lasseter R., Akhil A., Mamy C., Stephens J., Dagle J., Guttromson R., Meliopoulous A.S., Yinger R., Eto J. (2003). Integration of Distributed Energy Resources. The CERTS Microgrid Concept. White Paper. https://certs.lbl.gov/publications/integration-distributed-energy.

[14] Anduaga J., Boyra M., Cobelo I., García E., Gil A., Jimeno J., Laresgoiti I., Oyarzabal J.M., Rodríguez R., Sánchez E. (2008). La Microrred, Una Alternativa de Futuro Para un Suministro Energético Integral.

[15] Hoy M.B. (2016). Smart buildings: An introduction to the library of the future. Med. Ref. Serv. Q..

[16] Fang X., Misra S., Xue G., Yang D. (2012). Smart grid—The new and improved power grid: A Survey. IEEE Commun. Surv. Tutor..

[17] Singh A., Nasiruddin I., Khatoon S., Muazzam M., Chaturvedi D.K. Load Forecasting Techniques and Methodologies: A Review. Proceedings of the 2nd International Conference on Power, Control and Embedded Systems (ICPCES).

[18] Nti I.K., Teimeh M., Nyarko-Boateng O., Adekoya A.F. (2020). Electricity load forecasting: A systematic review. J. Electr. Syst. Inf. Technol..

[19] Ahmed Mir A., Alghassab Kafait M., Ullah K., Ali Khan Z., Lu Y., Imran M. (2020). A review of electricity demand forecasting in low and middle income countries: The demand determinants and horizons. Sustainability.

[20] Clements A.E., Hurn A.S., Li Z. (2016). Forecasting day-ahead electricity load using a multiple equation time series approach. Eur. J. Oper. Res..

[21] Hong Y., Zhou Y., Li Q., Xu W., Zheng X. (2020). A deep learning method for short-term residential load forecasting in smart grid. IEEE Access.

[22] Phuangpornpitak N., Prommee W. (2016). A study of load demand forecasting models in electric power system operation and planning. GMSARN Int. J..

[23] Xiao L., Shao W., Liang T., Wang C. (2016). A combined model based on multiple seasonal patterns and modified firefly algorithm for electrical load forecasting. Appl. Energy.

[24] Xue B., Geng J. Dynamic Transverse Correction Method of Middle and Long-term Energy Forecasting based on Statistic of Forecasting Errors. Proceedings of the 10th Conference on Power and Energy (IPEC).

[25] Khatoon S., Nasiruddin I., Singh A., Gupta P. Effects of Various Factors on Electric Load Forecasting: An Overview. Proceedings of the IEEE Power India International Conference (PIICON).

[26] Hippert H.S., Pedreira C.E., Souza R.C. (2001). Neural networks for short-term load forecasting: A review and evaluation. IEEE Trans. Power Syst..

[27] Fahad M.U., Arbab N. (2014). Factor affecting short term load forecasting. J. Clean Energy Technol..

[28] Novianto D., Gao W., Kuroki S. (2015). Review on people’s lifestyle and energy consumption of Asian communities: Case study of Indonesia, Thailand, and China. Energy Power Eng..

[29] Hernández L., Baladrón C., Aguiar J.M., Calavia L., Carro B., Sánchez-Esguevillas A., García P., Lloret J. (2013). Experimental analysis of the input variables’ relevance to forecast next day’s aggregated electric demand using neural networks. Energies.

[30] Issi F., Kaplan O. (2018). The determination of load profiles and power consumptions of home appliances. Energies.

[31] Shi Y., Yu T., Liu Q., Zhu H., Li F., Wu Y. (2020). An approach of electrical load profile analysis based on time series data mining. IEEE Access.

[32] Hafeez G., Alimgeer K.S., Khan I. (2020). Electric load forecasting based on deep learning and optimized by heuristic algorithm in smart grid. Appl. Energy.

[33] Gillies D.K.A., Bernholtz B., Sandiford P.J. (1956). A new approach to forecasting daily peak loads. Trans. Am. Inst. Electr. Eng. Part III Power Appar. Syst..

[34] Park D.C., El-Sharkawi M.A., Marks II R.J., Atlas L.E., Damborg M.J. (1991). Electric load forecasting using an artificial neural network. IEEE Trans. Power Syst..

[35] Bakirtzis A.G., Petridis V., Kiartzis S.J., Alexiadis M.C., Maissis A.H. (1995). A neural network short term load forecasting model for the Greek power system. IEEE Trans. Power Syst..

[36] Lu C.-N., Wu H.-T., Vemuri S. (1993). Neural network based short term load forecasting. IEEE Trans. Power Syst..

[37] Papalexopoulos A.D., Hao S., Peng T.-M. (1994). An implementation of a neural network based load forecasting models for the EMS. IEEE Trans. Power Syst..

[38] Wang P. (2019). On defining artificial intelligence. J. Artif. Gen. Intell..

[39] Ongsulee P. Artificial Intelligence, Machine Learning and Deep Learning. Proceedings of the 15th International Conference on ICT and Knowledge Engineering (ICT&KE).

[40] Raz A.R., Llinas J., Mittu R., Lawless W.F., Lawless W.F., Mittu R., Sofge D.A. (2020). Engineering for emergence in information fusion systems: A review of some challenges. Human-Machine Shared Contexts.

[41] Cohen S., Cohen S. (2021). The basics of machine learning: Strategies and techniques. Artificial Intelligence and Deep Learning in Pathology.

[42] Bonetto R., Latzko V., Fitzek F.H.P., Granelli F., Seeling P. (2020). Machine learning. Computing in Communication Networks, From Theory to Practice.

[43] Delua J. (2021). Supervised vs. Unsupervised Learning: What’s the Difference?. https://www.ibm.com/cloud/blog/supervised-vs-unsupervised-learning.

[44] Sarker I.H. (2021). Machine learning: Algorithms, real-world applications and research directions. SN Comput. Sci..

[45] Choi R.Y., Coyner A.S., Kalpathy-Cramer J., Chiang M.F., Campbell J.P. (2020). Introduction to machine learning, neural networks, and deep learning. Transl. Vis. Sci. Technol..

[46] Alloghani M., Al-Jumeily D., Mustafina J., Hussain A., Aljaaf A.J., Berry M., Mohamed A., Yap B. (2020). A systematic review on supervised and unsupervised machine learning algorithms for data science. Supervised and Unsupervised Learning for Data Science.

[47] Satinet C., Fouss F. (2022). A supervised machine learning classification framework for clothing products’ sustainability. Sustainability.

[48] Jiang T., Gradus J.L., Rosellini A.J. (2020). Supervised machine learning: A brief primer. Behav. Ther..

[49] El Bouchefry K., de Souza R.S., Škoda P., Adam F. (2020). Learning in big data: Introduction to machine learning. Knowledge Discovery in Big Data from Astronomy and Earth Observation.

[50] Uddamari N., Ubbana J. (2021). A study on unsupervised learning algorithms analysis in machine learning. Turk. J. Comput. Math. Educ..

[51] Khanum M., Mahboob T., Imtiaz W., Ghafoor H., Sehar R. (2015). A survey on unsupervised machine learning algorithms for automation, classification and maintenance. Int. J. Comput. Appl..

[52] Avinash K., William G., Ryan B. Network Attack Detection Using an Unsupervised Machine Learning Algorithm. Proceedings of the Hawaii International Conference on System Sciences (HICSS).

[53] Chen Y., Kong R., Kong L., Kong L., Huang T., Zhu Y., Yu S. (2020). Applications of artificial intelligence in astronomical big data. Big Data in Astronomy.

[54] Van Engelen J.E., Hoos H.H. (2020). A survey on semi-supervised learning. Mach. Learn..

[55] ZhongKaizhu G., Huang H. (2018). Semisupervised Learning: Background, Applications and Future Directions.

[56] Huynh T., Nibali A., He Z. (2022). Semisupervised learning for medical image classification using imbalanced training data. Comput. Methods Programs Biomed..

[57] Kaur A., Gourav K. (2020). A study of reinforcement learning applications & its algorithms. Int. J. Sci. Technol. Res..

[58] Mohammed M., Khan M.B., Bashier Mohammed B.E. (2016). Machine Learning: Algorithms and Applications.

[59] Tanveer J., Haider A., Ali R., Kim A. (2022). An overview of reinforcement learning algorithms for handover management in 5G ultra-dense small cell networks. Appl. Sci..

[60] Liu S., See K.C., Ngiam K.Y., Celi L.A., Sun X., Feng M. (2020). Reinforcement learning for clinical decision support in critical care: Comprehensive review. J. Med. Internet Res..

[61] Hao X., Zhang G., Ma S. (2016). Deep learning. Int. J. Semant. Comput..

[62] Nichols J.A., Herbert Chan H.W., Baker M. (2019). Machine learning: Applications of artificial intelligence to imaging and diagnosis. Biophys. Rev..

[63] Sadiq R., Rodríguez M.J., Mian H.R., Nriagu J. (2019). Empirical models to predict Disinfection By-Products (DBPs) in drinking water: An updated review. Encyclopedia of Environmental Health.

[64] Teuwen J., Moriakov N., Zhou S.K., Rueckert D., Fichtinger G. (2020). Convolutional neural networks. Handbook of Medical Image Computing and Computer Assisted Intervention.

[65] He Z. Deep Learning in Image Classification: A Survey Report. Proceedings of the 2nd International Conference on Information Technology and Computer Application (ITCA).

[66] Khan A., Sohail A., Zahoora U., Qureshi A.S. (2020). A survey of the recent architectures of deep convolutional neural networks. Artif. Intell. Rev..

[67] Cho K., van Merrienboer B., Bahdanau D., Bengio Y. (2014). On the Properties of Neural Machine Translation: Encoder-Decoder Approaches. arXiv.

[68] Elman J. (1990). Finding structure in time. Cogn. Sci..

[69] Toha S.F., Tokhi M.O. MLP and Elman Recurrent Neural Network Modelling for the TRMS. Proceedings of the 7th IEEE International Conference on Cybernetic Intelligent Systems.

[70] Liu Y., Guo J., Cai C., Wang Y., Jia L. Short-Term Forecasting of Rail Transit Passenger Flow Based on Long Short-Term Memory Neural Network. Proceedings of the International Conference on Intelligent Rail Transportation (ICIRT).

[71] Zhao Z., Liang Y., Jin X. Handling Large-Scale Action Space in Deep Q Network. Proceedings of the International Conference on Artificial Intelligence and Big Data (ICAIBD).

[72] Xie W., Ouyang Y., Ouyang J., Rong W., Xiong Z. User Occupation Aware Conditional Restricted Boltzmann Machine Based Recommendation. Proceedings of the IEEE International Conference on Internet of Things (iThings) and IEEE Green Computing and Communications (GreenCom) and IEEE Cyber, Physical and Social Computing (CPSCom) and IEEE Smart Data (SmartData).

[73] Taylor G.W., Hinton G.E., Roweis S.T. (2011). Two distributed-state models for generating high-dimensional time series. J. Mach. Learn. Res..

[74] Hinton G.E., Osindero S., Teh Y.W. (2006). A fast learning algorithm for deep belief nets. Neural Comput..

[75] Bedi G., Venayagamoorthy G.K., Singh R. (2020). Development of an IoT-driven building environment for prediction of electric energy consumption. IEEE Internet Things J..

[76] Timur O., Zor K., Çelik Ö., Teke A., İbrikçi T. (2020). Application of statistical and artificial intelligence techniques for medium-term electrical energy forecasting: A case study for a regional hospital. J. Sustain. Dev. Energy Water Environ. Syst..

[77] Selvi M.V., Mishra S. Investigation of Weather Influence in Day-Ahead Hourly Electric Load Power Forecasting with New Architecture Realized in Multivariate Linear Regression Artificial Neural Network Techniques. Proceedings of the 8th IEEE India International Conference on Power Electronics (IICPE).

[78] Selvi M.V., Mishra S. (2020). Investigation of performance of electric load power forecasting in multiple time horizons with new architecture realized in multivariate linear regression and feed-forward neural network techniques. IEEE Trans. Ind. Appl..

[79] Eseye A.T., Lehtonen M., Tukia T., Uimonen S., Millar R.J. (2019). Machine learning based integrated feature selection approach for improved electricity demand forecasting in decentralized energy systems. IEEE Access.

[80] Wen L., Zhou K., Yang S. (2020). Load demand forecasting of residential buildings using a deep learning model. Electr. Power Syst. Res..

[81] Rodríguez F., Galarza A., Vasquez J.C., Guerrero J.M. (2022). Using deep learning and meteorological parameters to forecast the photovoltaic generators intra-hour output power interval for smart grid control. Energy.

[82] Taleb I., Guerard G., Fauberteau F., Nguyen N.A. (2022). Flexible deep learning method for energy forecasting. Energies.

[83] Xu A., Tian M.-W., Firouzi B., Alattas K.A., Mohammadzadeh A., Ghaderpour E. (2022). A new deep learning Restricted Boltzmann Machine for energy consumption forecasting. Sustainability.

[84] Yem Souhe F.G., Franklin Mbey C., Teplaira Boum A., Ele P., Foba Kakeu V.J. (2022). A hybrid model for forecasting the consumption of electrical energy in a smart grid. J. Eng..

[85] Aurangzeb K., Alhussein M., Javed K., Haider S.I. (2021). A Pyramid-CNN based deep learning model for power load forecasting of similar-profile energy customers based on clustering. IEEE Access.

[86] Jahangir H., Tayarani H., Gougheri S.S., Golkar M.A., Ahmadian A., Elkamel A. (2021). Deep learning-based forecasting approach in smart grids with microclustering and bidirectional LSTM network. IEEE Trans. Ind. Electron..

[87] Mubashar R., Awan M.J., Ahsan M., Yasin A., Singh V.P. (2022). Efficient residential load forecasting using deep learning approach. Int. J. Comput. Appl. Technol..

[88] Rosato A., Araneo R., Andreotti A., Panella M. 2-D Convolutional Deep Neural Network for Multivariate Energy Time Series Prediction. Proceedings of the 2019 IEEE International Conference on Environment and Electrical Engineering and 2019 IEEE Industrial and Commercial Power Systems Europe (EEEIC/I&CPS Europe).

[89] Zhang X., Biagioni D., Cai M., Graf P., Rahman S. (2020). An edge-cloud integrated solution for buildings demand response using reinforcement learning. IEEE Trans. Smart Grid.

[90] Aurangzeb K., Alhussein M. Deep Learning Framework for Short-term Power Load Forecasting, a Case Study of Individual Household Energy Customer. Proceedings of the 2019 International Conference on Advances in the Emerging Computing Technologies (AECT).

[91] Escobar E., Rodríguez Licea M.A., Rostro-Gonzalez H., Espinoza Calderon A., Barranco Gutiérrez A.I., Pérez-Pinal F.J. Comparative Analysis of Multivariable Deep Learning Models for Forecasting in Smart Grids. Proceedings of the 2020 IEEE International Autumn Meeting on Power, Electronics and Computing (ROPEC).

[92] Hafeez G., Alimgeer K.S., Wadud Z., Shafiq Z., Ali Khan M.U., Khan I., Khan F.A., Derhab A. (2020). A novel accurate and fast converging deep learning-based model for electrical energy consumption forecasting in a smart grid. Energies.

[93] Nguyen V.-B., Duong M.-T., Le M.-H. Electricity Demand Forecasting for Smart Grid based on Deep Learning Approach. Proceedings of the 2020 5th International Conference on Green Technology and Sustainable Development (GTSD).

[94] Rosato A., Succetti F., Araneo R., Andreotti A., Mitolo M., Panella M. A Combined Deep Learning Approach for Time Series Prediction in Energy Environments. Proceedings of the 2020 IEEE/IAS 56th Industrial and Commercial Power Systems Technical Conference (I&CPS).

[95] Qi X., Zheng X., Chen Q. A Short-term Load Forecasting of Integrated Energy System based on CNN-LSTM. Proceedings of the 2020 International Conference on Energy, Environment and Bioengineering (ICEEB 2020).

[96] Wang B., Li Y., Ming W., Wang S. (2020). Deep reinforcement learning method for demand response management of interruptible load. IEEE Trans. Smart Grid.

[97] Wen L., Zhou K., Li J., Wang S. (2020). Modified deep learning and reinforcement learning for an incentive-based demand response model. Energy.

[98] Yang Y., Li W., Gulliver T.A., Li S. (2020). Bayesian deep learning-based probabilistic load forecasting in smart grids. IEEE Trans Ind. Inf..

[99] Amin P., Cherkasova L., Aitken R., Kache V. Automating Energy Demand Modeling and Forecasting Using Smart Meter Data. Proceedings of the 2019 IEEE International Congress on Internet of Things (ICIOT).

[100] Atef S., Eltawil A.B. A Comparative Study Using Deep Learning and Support Vector Regression for Electricity Price Forecasting in Smart Grids. Proceedings of the IEEE 6th International Conference on Industrial Engineering and Applications (ICIEA).

[101] Chan S., Oktavianti I., Puspita V. A Deep Learning CNN and AI-Tuned SVM for Electricity Consumption Forecasting: Multivariate Time Series Data. Proceedings of the 2019 IEEE 10th annual information technology, Electronics and Mobile Communication Conference (IEMCON).

[102] Hafeez G., Javaid N., Riaz M., Ali A., Umar K., Iqbal Q.Z. Day Ahead Electric Load Forecasting by an Intelligent Hybrid Model based on Deep Learning for Smart Grid. Proceedings of the 14th International Conference on Complex, Intelligent and Software Intensive Systems (CISIS-2020).

[103] Kaur D., Kumar R., Kumar N., Guizani M. Smart Grid Energy Management using RNN-LSTM: A Deep Learning-Based Approach. Proceedings of the IEEE Global Communications Conference (GLOBECOM).

[104] Al Khafaf N., Jalili M., Sokolowski P. (2019). Application of deep learning long short-term memory in energy demand forecasting. Commun. Comput. Inf. Sci..

[105] Khan A.B.M., Khan S., Aimal S., Khan M., Ruqia B., Javaid N., Barolli L., Xhafa F., Hussain O. Half hourly electricity load forecasting using convolutional neural network. Innovative Mobile and Internet Services in Ubiquitous Computing.

[106] Kim T.Y., Cho S.B. (2019). Predicting residential energy consumption using CNN-LSTM neural networks. Energy.

[107] Kim T., Cho S. Particle Swarm Optimization-based CNN-LSTM Networks for Forecasting Energy Consumption. Proceedings of the 2019 IEEE Congress on Evolutionary Computation (CEC).

[108] Lu R., Hong S.H. (2019). Incentive-based demand response for smart grid with reinforcement learning and deep neural network. Appl. Energy.

[109] Pramono S.H., Rohmatillah M., Maulana E., Hasanah R.N., Hario F. (2019). Deep learning-based short-term load forecasting for supporting demand response program in hybrid energy system. Energies.

[110] Rahman S., Alam M.G.R., Rahman M.M. Deep Learning based Ensemble Method for Household Energy Demand Forecasting of Smart Home. Proceedings of the 2019 22nd International Conference on Computer and Information Technology (ICCIT).

[111] Syed D., Refaat S.S., Abu-Rub H., Bouhali O., Zainab A., Xie L. Averaging Ensembles Model for Forecasting of Short-Term Load in Smart Grids. Proceedings of the IEEE International Conference on Big Data (Big Data).

[112] Ustundag Soykan E., Bilgin Z., Ersoy M.A., Tomur E. Differentially Private Deep Learning for Load Forecasting on Smart Grid. Proceedings of the 2019 IEEE Globecom Workshops (GC Wkshps).

[113] Vesa A.V., Ghitescu N., Pop C., Antal M., Cioara T., Anghel I.A., Salomie I. Stacking Ulti-Learning Ensemble Model for Predicting Near Real Time Energy Consumption Demand of Residential Buildings. Proceedings of the 2019 IEEE 15th International Conference on Intelligent Computer Communication and Processing (ICCP).

[114] Yang Y., Hong W., Li S. (2019). Deep ensemble learning based probabilistic load forecasting in smart grids. Energy.

[115] Zahid M., Ahmed F., Javaid N., Abbasi R.A., Zainab Kazmi H.S., Javaid A., Bilal M., Akbar M., Ilahi M. (2019). Electricity price and load forecasting using enhanced convolutional neural network enhanced support vector regression in smart grids. Electronics.

[116] Ouyang T., He Y., Li H., Sun Z., Baek S. (2019). Modeling and forecasting short-term power load with copula model and deep belief network. IEEE Trans. Emerg. Top. Comput. Intell..

[117] Hafeez G., Javaid N., Ullah S., Iqbal Q.Z., Khan M., Rehman A.U., Ullah Z. Short-Term Load Forecasting based on Deep Learning for Smart Grid Applications. Proceedings of the 12th International Conference on Innovative Mobile and Internet Services in Ubiquitous Computing (IMIS-2018).

[118] Koprinska I., Wu D., Wang Z. Convolutional Neural Networks for Energy Time Series Forecasting. Proceedings of the 2018 International Joint Conference on Neural Networks (IJCNN).

[119] Kuo P.H., Huang C.J. (2018). A high precision artificial neural networks model for short-Term energy load forecasting. Energies.

[120] Shi H., Xu M., Li R. (2018). Deep learning of household load forecasting—A novel pooling deep RNN. IEEE Trans Smart Grid.

[121] Ghaderi A., Sanandaji B.M., Ghaderi F. Deep forecast: Deep learning-based spatio-temporal forecasting. arXiv.

[122] Jarábek T., Laurinec P., Lucká M. Energy Load Forecast using S2S Deep Neural Networks with k-Shape Clustering. Proceedings of the 2017 IEEE 14th International Scientific Conference on Informatics 2017.

[123] Li L., Ota K., Dong M. Everything is Image: CNN-Based Short-Term Electrical Load Forecasting for Smart Grid. Proceedings of the 2017 14th International Symposium on Pervasive Systems, Algorithms and Networks & 2017 11th International Conference on Frontier of Computer Science and Technology & 2017 Third International Symposium of Creative Computing (ISPAN-FCST-ISCC).

[124] Zhan J., Huang J., Niu L., Peng X., Deng D., Cheng S. Study of the Key Technologies of Electric Power Big Data and Its Application Prospects in Smart Grid. Proceedings of the IEEE PES Asia-Pacific Power and Energy Engineering Conference (APPEEC).

